# Anticipatory prescribing of injectable medications for adults at the end of life in the community: A systematic literature review and narrative synthesis

**DOI:** 10.1177/0269216318815796

**Published:** 2018-12-04

**Authors:** Ben Bowers, Richella Ryan, Isla Kuhn, Stephen Barclay

**Affiliations:** 1 Department of Public Health and Primary Care, University of Cambridge, Cambridge, UK; 2 Medical Library, University of Cambridge, Cambridge, UK

**Keywords:** Anticipatory prescribing, palliative medicine kit, terminal care, palliative care, palliative medicine, review, systematic review, death

## Abstract

**Background:**

The anticipatory prescribing of injectable medications to provide end-of-life symptom relief is an established community practice in a number of countries. The evidence base to support this practice is unclear.

**Aim:**

To review the published evidence concerning anticipatory prescribing of injectable medications for adults at the end of life in the community.

**Design:**

Systematic review and narrative synthesis. Registered in PROSPERO: CRD42016052108, on 15 December 2016 (https://www.crd.york.ac.uk/prospero/display_record.php?RecordID=52108).

**Data sources:**

Medline, CINAHL, Embase, PsycINFO, Web of Science, Cochrane Library, King’s Fund, Social Care Online, and Health Management Information Consortium databases were searched up to May 2017, alongside reference, citation, and journal hand searches. Included papers presented empirical research on the anticipatory prescribing of injectable medications for symptom control in adults at the end of life. Research quality was appraised using Gough’s ‘Weight of Evidence’ framework.

**Results:**

The search yielded 5099 papers, of which 34 were included in the synthesis. Healthcare professionals believe anticipatory prescribing provides reassurance, effective symptom control, and helps to prevent crisis hospital admissions. The attitudes of patients towards anticipatory prescribing remain unknown. It is a low-cost intervention, but there is inadequate evidence to draw conclusions about its impact on symptom control and comfort or crisis hospital admissions.

**Conclusion:**

Current anticipatory prescribing practice and policy is based on an inadequate evidence base. The views and experiences of patients and their family carers towards anticipatory prescribing need urgent investigation. Further research is needed to investigate the impact of anticipatory prescribing on patients’ symptoms and comfort, patient safety, and hospital admissions.

**What is already known about the topic?**

- The anticipatory prescribing of injectable medications for adults at their end of life is recommended practice in a number of countries.
- Practitioners believe that anticipatory prescribing has a key role in ensuring patients receive effective and timely symptom control and in avoiding crisis inpatient admissions.

**What the paper adds?**

- Practice and policy are based on healthcare professionals’ views that anticipatory prescribing is reassuring to patients and their family carers and is clinically effective in providing effective symptom control.
- No studies have explored patients’ views and experiences of anticipatory prescribing
- Anticipatory prescribing is a low-cost intervention, but there is inadequate evidence to allow conclusions to be drawn about its cost-effectiveness, safety, impact on patient-reported symptoms, and comfort or prevention of crisis hospital admissions.

**Implications for practice, theory, or policy**

- Research is needed to investigate the impact of anticipatory prescribing on patient-reported symptom control and comfort, patient safety, and crisis hospital admission avoidance.
- The acceptability of anticipatory prescribing for patients and their family carers requires urgent investigation.

## Introduction

The management of pain, distress, and other symptoms at the end of life is a shared goal for patients, their family carers, and healthcare professionals.^1–5^ To meet the needs of patients approaching the end of their lives in the community, anticipatory prescribing has been promoted to optimise symptom control and prevent crisis hospital admissions.^6–10^ Anticipatory prescribing is the prescription and dispensing of injectable medications to a named patient, in advance of clinical need, for administration by suitably trained individuals if symptoms arise in the final days of life.^6,11^ Injectable medications are typically prescribed for four common symptoms: pain, nausea and vomiting, agitation, and respiratory secretions.^7,11,12^

Community-based anticipatory prescribing practices vary between countries based on local healthcare conventions, financial costs, legislation surrounding controlled drugs, and the availability of healthcare professionals to administer medications when needed.^9,13–15^ Studies in the United States of America^15–17^ and Singapore^9^ report on schemes where drugs are prescribed in oral, sublingual, or rectal forms for family members to administer. In the United Kingdom^7,8^ and Australia,^18^ it is considered good practice to prescribe and dispense injectable medications that offer reliable and rapid symptom relief when patients can no longer manage oral medications during the dying phase.^11^ They are typically administered by nurses or general practitioners (GPs) based on their clinical assessment that the person is dying and has irreversible symptoms.^3,7,19,20^

Anticipatory prescribing of injectable medication in the community first appeared in the literature by Amass and Allen^6^ and has subsequently been adopted as a central component of good end-of-life care planning in the United Kingdom, Australia, and New Zealand.^7,10,11,18,21^ Anticipatory prescribing is recommended to follow an individualised approach after assessment of a patient’s particular needs and situation.^7,22^ It ensures rapid access to medications, particularly out-of-hours when sourcing medication can be delayed^22–25^ and enables rapid administration of drugs when out-of-hours clinicians may have limited knowledge of a patient’s situation.^26^

However, prescribing strong injectable medications ahead of need has potential risks. Appropriate prescribing relies on GPs correctly identifying that patients are approaching their last days of life.^3,24,27^ Appropriate administration is dependent on nurses correctly diagnosing that symptoms are not reversible and that the patient is dying; a skilled judgement requiring multidisciplinary discussion with senior colleagues in the hospital setting.^19^ The prescriber remains accountable for the drugs, including strong opioids, which may be in the home for weeks^7^ and are open to misuse by visitors and family members.^3,28^ In the United Kingdom, the critical review of the Liverpool Care Pathway found that the use of anticipatory prescribing without adequate explanation or justification led to families being concerned about over-sedation and drugs hastening death.^29^

Despite these concerns, subsequent UK end-of-life care guidance continues to advocate individualised anticipatory prescribing as best practice.^7,8^ However, the same guidance^7^ highlighted the limited evidence base concerning anticipatory prescribing practice and the risk that drugs are sometimes prescribed in a ‘blanket-like fashion’ rather than tailored to patients’ needs.

In summary, it is unclear whether anticipatory prescribing is acceptable to all involved, clinically effective or cost-effective.

## Aim

It was, therefore, decided to undertake a systematic literature review concerning anticipatory prescribing for adults at the end of life in the community. The focus is exclusively on injectable medications, as this is the most widespread form of anticipatory prescribing, requires specific training, and has been highlighted to have potential for misuse.^7,29^

## Review questions

With regard to anticipatory prescribing of injectable medications for adults in the community approaching the end of their lives:

1. What is current practice?
2. What are the attitudes of patients?
3. What are the attitudes of family carers?
4. What are the attitudes of community healthcare professionals?
5. What is its impact on patient comfort and symptom control?
6. Is it cost-effective?

## Methods

### Eligibility criteria

Papers were included if they presented empirical research on the anticipatory prescribing of injectable medications for symptom control in adults (aged 18 years and over) at the end of life in the community. Box 1 presents detailed inclusion and exclusion criteria.

**Box 1.** Inclusion and exclusion criteria.

Inclusion criteria: Published papers presenting empirical research on the prescribing of injectable medications ahead of need to control terminal symptoms for adults (aged 18 years and over). Participants receiving care at home in the community (including nursing and residential home care settings). Peer-reviewed quantitative and qualitative studies, case studies, audits, and published conference abstracts. Key areas for data extraction: Descriptions of current practice; Patient-reported acceptance and views; Family carer–reported acceptance and views; Healthcare professional–reported acceptance and views; Patient comfort/symptom control (reported by whom); Evidence for cost-effectiveness, including impact on: - Admission avoidance; - Place of death; - Healthcare activity; - Cost of drugs. Studies published up until May 2017. English language full text. Exclusion criteria: Anticipatory prescribing in non-terminal care situations. Prescriptions that do not include injectable medication. Children (aged 17 years or under). Prescribing in hospital, hospice, or prisons. Papers with no new empirical data, for example, editorials, opinion papers, or narrative reviews. Research examining assisted dying or euthanasia. Research examining continuous sedation until death. Studies concerning administration of medication via continuous subcutaneous infusion (syringe driver). Grey literature.

### Search strategy

The search strategy was developed in collaboration with a specialist information technologist (I.K.). The search strategy in Medline is presented in Box 2 and was adapted for each subsequent database (CINAHL, Embase, PsycINFO, Web of Science, Cochrane Library, King’s Fund, Social Care Online and Health Management Information Consortium (HMIC); **Supplemental document 1**). All databases were searched from inception to May 2017. In addition, Palliative Medicine and British Medical Journal Supportive and Palliative Care were hand-searched from January 2007 to May 2017. Reference and citation searches of all included papers were undertaken.

**Box 2.** Medline search strategy.

Epub Ahead of Print, In-Process & Other Non-Indexed Citations, Ovid MEDLINE(R) Daily and Ovid MEDLINE(R) 1946 to Present ((palliative adj medicine adj kit*) or (liverpool adj care adj pathway*) or ((end adj2 life) adj2 ((care adj plan*) or (care adj pathway*))) or (gold adj standard* adj framework*) or ((prescrib* or prescription* or medicat* or medicine* or drug* or pharma or pharmaceutical* or packet* or pack* or pak* or box* or kit* or (care adj plan*) or (core adj ‘4’) or (core adj four)) adj3 (crisis* or comfort* or anticipate* or anticipatory or anticipation or preemptive or pre-emptive or (just adj in adj case) or PRN or (pro adj re adj nata) or (as adj required)))).ti, ab. AND (exp Terminal Care/ or exp Palliative Care/ or exp ‘Hospice and Palliative Care Nursing’/ or exp death/ or exp Palliative Medicine/ or exp Terminally Ill/ or ((end adj2 life) or ((final* or last*) adj1 (hour* or day* or minute* or week* or month* or moment*)) or palliat* or terminal* or (end adj stage) or dying or (body adj2 (shutdown or shut* down or deteriorat*)) or deathbed).ti, ab.) Searches in CINHAL, Embase, PsycINFO, Web of Science, Cochrane Library, King’s Fund, Social Care Online and HMIC were adapted from this strategy. The full search strategy is available in ‘**Supplemental Document 1**’.

### Study selection

**Figure 1.**
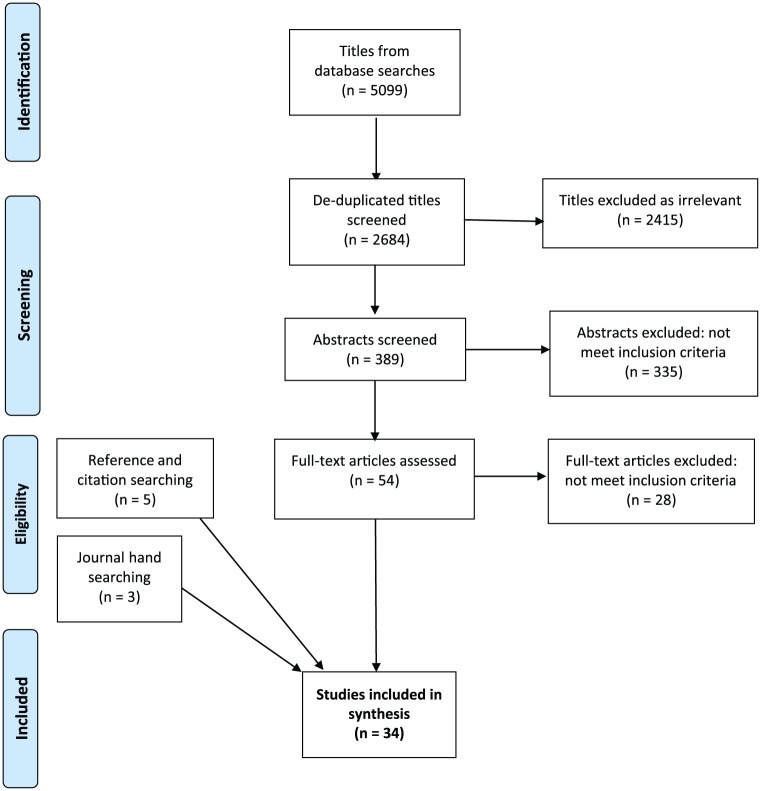
PRISMA flow diagram.

After exclusion of irrelevant and duplicate titles, abstracts were screened for eligibility independently by two reviewers (B.B. and R.R.) with disagreement between reviewers resolved by consensus. Full-texts of all potentially relevant papers were then assessed for eligibility by B.B. and with a second review by R.R. where eligibility was uncertain (Figure 1).

### Data extraction, quality appraisal, and data synthesis

A review-specific data extraction form was designed and piloted on five papers. Two reviewers (B.B. and R.R.) then independently extracted data from each eligible paper: publication details, study aims, participants, methods, and results relevant to each of the six review questions (**Supplemental Document 2**).

Two reviewers (B.B. and R.R.) then independently critically appraised the quality and relevance of each included study using Gough’s ‘Weight of Evidence’ (WoE) framework^30^ (Table 1). This framework rates both the quality and relevance of included studies using four domains of assessment concerning the internal validity of the study (WoE A), the appropriateness of study design to the review aims (WoE B) and the focus or relevance of the study to the review aims (WoE C). These three domains were then combined into an overall judgement of study quality and relevance (WoE D). Where the reviewer was also an author of a selected study, a third reviewer (S.B.) conducted the quality assessment. Discrepancies in quality appraisal decisions were discussed and consensus achieved.

**Table 1.** Review-specific Gough’s ‘Weight of Evidence’ criteria.

WoE A was judged against internal validity: whether the study design was rigorous; whether this could be adequately assessed from a transparent, comprehensive, and repeatable method; accurate and understandable presentation and analysis; if samples and data collection tools were appropriate to the aims of the study and whether conclusions flowed from the findings and are proportionate to the method. Papers were scores as high/medium/low.
WoE B relates to the appropriateness of the study design to the six review-specific questions. Papers were scores as high/medium/low. Review questions 2, 3, and 4: inductive research designs interpreting the views directly reported by patients/carers/healthcare professionals = high. Deductive research designs interpreting the views directly reported by patients/family carers/healthcare professionals = medium. Deductive research designs indirectly interpreting the views of patients/family carers/healthcare professionals = low. Review questions 1, 5, and 6: the fitness for purpose of the study design in answering the questions were made on a paper-by-paper basis.
WoE C relates to detailed judgements about each study relating to the relevance of the focus of the evidence for answering the review questions. This includes: consideration of any sampling issues relating to the interpretation of the data; whether the study was undertaken in an appropriate context from which results can be generalised to answer the relevant review-specific questions. Papers were scores as high/medium/low.
Weight of Evidence D (WoE D): the above three sets of judgement scores are then combined to give the overall ‘weight of evidence’ as high/medium/low.

The criteria given in the table are adapted from a study by Gough.^30^

Data synthesis used a narrative approach.^31,32^ This was chosen for its applicability to the synthesis of a range of qualitative and quantitative evidence.^32^ The narrative synthesis involved the following three iterative stages:

1. Developing a preliminary synthesis: B.B. created a textual description of each study from the data extraction forms. Study descriptions were grouped together and tabulated based on the sample population and the research questions the results answered. B.B. carried out an inductive thematic analysis to identify the main, recurrent, and important data across the studies in answering each research question.^31,32^
2. Exploring relationships in the data: B.B. (a nurse researcher) and R.R. (a palliative doctor and clinical academic) constructed the interpretive synthesis by independently reviewing the thematic analysis and exploring heterogeneity across studies.^31,32^ Particular attention was placed on the differences and similarities between the studies, including methodological approaches, context, the characteristics of the populations being studied, and results. The results which emerged from studies conducted by researchers from different disciplinary and epistemological positions were debated and consensus in the synthesis was reached.^32^ The synthesis was further refined through discussion of the review results and their implications with clinicians, interdisciplinary academic audiences, and S.B. (a GP and clinical academic).
3. Assessing the robustness of the synthesis: the quality and relevance assessment using Gough’s WoE framework^30^ informed each stage of the synthesis. Papers judged as being of high quality using Gough’s WoE framework were considered more credible and relevant than medium quality papers throughout data synthesis.^30,32^ Conclusions drawn only from papers assessed under WoE D to be of low quality were deemed inadequate unless they supported the findings of high or medium quality papers.^32^ The reviewers decided to include low quality evidence in the synthesis to demonstrate that current anticipatory prescribing practice is largely based on low and medium quality evidence, highlighting the gaps in knowledge and the need for future research (Table 2).

**Table 2.** Number of papers included in the synthesis.

Review question	Number of papers answering each review question
What is current practice?	26 papers: 3 high, 16 medium, 7 low quality
What are the attitudes of patients?	No papers on patient views or experiences. 2 papers refer to practitioner interpretations of patient views: 1 medium and 1 low quality
What are the attitudes of family carers?	5 papers: 2 medium and 3 low quality
What are the attitudes of community healthcare professionals?	21 papers: 3 high, 13 medium, and 5 low quality
What is its impact on patient comfort and symptom control	3 papers: 2 medium and 1 low quality
Is it cost-effective?	9 papers: 6 medium and 3 low quality

The review protocol was registered with PROSPERO (reg. no. 42016052108).

## Results

The paper identification process is summarised in Figure 1. Database searches identified 2684 titles after de-duplication: journal hand searches identified three conference abstracts with five papers from reference and citation searching. A total of 34 papers, reporting on 30 studies, were included in the synthesis: 24 research papers and 10 conference abstracts. Two studies were reported in two papers^33–36^ and one study in three papers: ^19,20,37^ as each paper presented different findings, they were treated as individual study units in the synthesis. Papers reported on practice in the United Kingdom (n = 28), Australia (n = 5), and Canada (n = 1). Published papers’ methods included qualitative interviews with healthcare professionals (n = 15), qualitative interviews with family carers (n = 2), retrospective patient notes reviews (n = 7), staff or family carers questionnaires (n = 6), and clinical audits (n = 4). Table 3 summaries the included papers and their weighting on Gough’s WoE framework:^30^ 3 were rated high quality, 22 medium quality, and 9 low quality.

**Table 3.** Summary of included studies.

Author	Country	Participants	Aims	Research methods	Key findings	Weight of evidence A + B + C = D
Faull et al.^3^	United Kingdom	63 healthcare professionals working in one county: 22 GPs; 16 community nurses; 3 community pharmacists; 1 student Nurse; 4 community palliative care nurses; 17 community specialist nurses	To explore the issues that arise for practitioners working in the community, in relation to anticipatory prescribing for terminally ill patients who wish to die at home	Qualitative interviews and focus groups. Qualitative analysis	Participants valued the principle of anticipatory prescribing Decisions on when to prescribe more of an issue when non-cancer diagnosis It was uncommon to hear accounts of getting drugs in the home more than a day or two ahead of anticipated need Barriers to prescribing: potential drug waste, not knowing the patient well enough, concerns around prescriber accountability (especially opioids), situations where there may be drug misuse, and not knowing or trusting other professionals’ judgements Facilitators to prescribing: having known the patient for some time. Good communication between professionals	H H H – H
Wilson et al.^19^	United Kingdom	61 nurses working in two regions: 16 nursing home nurses; 27 community nurses; 18 community palliative care nurses 83 episodes of observations across 4 nursing homes and 4 community teams	To examine nurses’ decisions, aims, and concerns when using anticipatory medications	Ethnographic study using observations and qualitative interviews. Qualitative analysis	The aim expressed by nurses when using anticipatory medications was to ‘comfort’ and ‘settle’ dying patients and prevent admissions to hospital Nurses would only administer medication if symptoms that were both irreversible and due to entry into the dying phase, the patient consented (where possible) and was unable to take oral medication, and decisions were made independent of a patient’s relatives influence Nurses often worked in pairs to check prescriptions and aid decision-making Administering the medication raised a number of concerns: distinguish between pain and agitation so as to administer the most appropriate drug; not wanting to instigate administering drugs too soon; and balancing the risks of under-medicating against concerns about over-medicating and causing unwanted side effects Less experienced nurses expressed concerns about whether medications to control pain, particularly opioids, and symptoms hasten death. Concerns re ‘last injection’	H H H – H
Bowers and Redsell^24^	United Kingdom	11 nurses working in one county: 7 community palliative care nurses; 4 community nurses	To explore community nurses’ decision-making processes around the prescribing of anticipatory prescribing for people who are dying	Qualitative interviews. Qualitative analysis	Anticipatory medications represent a safety net and give nurses a sense of control in managing an individual’s last days of life symptom Management nurses felt that it was important to have medications to cover out-of-hour periods Nurses requested GPs prescribed drugs and negotiated with them over what drugs to prescribe facilitators to prescribing: keeping GPs up to date with the patient’s changing condition, good multidisciplinary communication, established relationship of mutual trust with GPs Barriers to prescribing: difficult to accurately predict when patients were likely to die; Some GPs worried that medications might be used inappropriately; Some GPs lacked up to date end-of-life drug knowledge and needed persuading to prescribe for all likely terminal symptoms	H M H – H
Rosenberg et al.^22^	Australia	18 family carers in one city	To examine the experiences of family caregivers supporting a dying person in the home setting, with particular regard to being supplied with an anticipatory prescribing kit	Qualitative interviews. Qualitative analysis	Patients are issued with anticipatory prescribing kits, and family carers are asked to administer injectable medications The introduction of the kit was viewed positively by most family carers Family carers found it reassuring that the kit improved accessibility should symptoms become difficult to control Some family carers were reluctant to give the medication and looked to nurses to administer drugs The expectation to administer medication was overwhelming and intimidating for some family carers	M H M – M
Finucane et al.^38^	United Kingdom	71 patients who died in eight nursing homes	To investigate the extent of anticipatory prescribing for residents who died in nursing homes in Lothian, Scotland	Retrospective notes review. Descriptive statistics	54% of residents who died in the nursing homes had a prescription for at least one anticipatory medicine 15% of residents had anticipatory prescriptions in place for all four common symptoms at the end of life There was great variation in anticipatory prescribing across the nursing homes: 100% of patients died with drugs prescribed in one nursing home compared with only 13% in another.	M M H – M
Perkins et al.^5^	United Kingdom	110 patients and 66 nurses and care staff in eleven nursing homes	To assess the impact of the Liverpool Care Pathway (LCP) on care in nursing homes and intensive care units	Mixed methods: retrospective case note review; 8 observations, linked with case note analysis; qualitative interviews with staff. Thematic analysis	Usually, when nursing home staff identified patients as being ‘weeks from death,’ they would request anticipatory prescribing Anticipatory prescribing was seen as a solution to problems with gaining timely medical input out of hours and avoidance of hospital admissions There was a strong emphasis in the nursing homes on being prepared for a patient’s death: anticipatory prescribing was viewed as essential Barriers to prescribing: GPs perceptions of the cost of wasted drugs; getting a timely review of the patient by the GP Most anticipatory medications went unused The administration of drugs often left nurses feeling uncomfortable, particularly if the patient died soon after their administration	H M M – M
Wilson and Seymour^37^	United Kingdom	72 healthcare professionals working in two regions: 61 nurses; 8 GPs; and 3 community pharmacists 83 episodes of observations	Aim not stated – reporting on a theme from a wider piece of research^19^	Ethnographic study using observations and qualitative interviews. Qualitative analysis	Nurses often initiated conversations with GPs about getting anticipatory prescribing in place. GPs were happy to take this advice Nursing participants reported that a small number of GPs were reluctant to prescribe anticipatory medications Barriers to prescribing: GPs did not regularly prescribe end-of-life drugs and lacked the confidence to do so without guidance, some nurses felt their expertise was not valued by GPs Facilitators to prescribing: trust, valuing each other’s knowledge and expertise, access to each other, and clarification of professional responsibilities comprise a central component of successful anticipatory prescribing	M H M – M
Brand et al.^39^	United Kingdom	12 healthcare professionals in one county: disciplines not stated	To explore the viewpoints of healthcare professionals involved in anticipatory prescribing in care homes	Qualitative interviews. Qualitative Thematic analysis analysis	Uncertainties surrounding when anticipatory prescribing should be initiated often results in residents not having drugs available until after symptoms appear Perception that anticipatory prescribing may reduce hospital admissions and provides symptom control Facilitators to prescribing: trusting relationships between professionals; good interdisciplinary communication	M M M – M
Brewerton et al.^40^	United Kingdom	150 patients accessing one community specialist palliative care service	To understand the current practice of anticipatory prescribing for patients referred to a community specialist palliative care service	Retrospective notes review. Descriptive statistics	63% had anticipatory prescribing. 55 of 100 patients with a cancer diagnosis had drugs in place verses 39 of 50 patients with a non-cancer diagnosis The median length of time from requesting anticipatory prescribing to death was 18 days 74 out of 97 patients who died in their preferred place of death had anticipatory prescribing	M M M – M
Griggs^41^	United Kingdom	17 community nurses within one county	To gain an insight into perceptions of a ‘good death’ among community nurses and to identify its central components	Qualitative interviews. Qualitative analysis	Nurses felt it was important to have drugs available ahead of need in homes Nurses were relied upon by GPs to recommend palliative drugs. Some Nurses did not like this responsibility Barriers to prescribing: perception that GPs reluctant to prescribe medications especially during out-of-hours periods	M M M – M
Israel et al.^23^	Australia	14 family caregivers in once city.	To investigate family caregivers perceptions of administering subcutaneous medications	Qualitative interviews. Qualitative analysis	All the family carers administered injectable anticipatory medications for at least 7 days Family carers felt they had no option but to give injections if their family member was to be cared for at home All placed a high value on the ability to contribute immediately to symptom control needs If symptoms were not controlled following injections, family carers felt disempowered and distressed Family carers expressed concern and uncertainty over timings of injections and feared causing medication overdose 2 family carers were concerned about the possibility of administering the ‘last injection’	M H L – M
Harris et al.^35^	United Kingdom	11 nurses from two different palliative care units and two head and neck wards: including 3 specialist palliative care nurses working in the community	To evaluate the utility of crisis medication in the management of terminal haemorrhage, through the experiences of nurses who have managed such events	Qualitative interviews. Interpretative phenomenological analysis	Participants’ experiences suggested that crisis medication had served little, if any, useful role in the management of terminal haemorrhage Challenging to know when to administer drugs and if events are reversible until it is too late	M H L – M
Harris et al.^36^	United Kingdom	8 nurses working in palliative care or head and neck setting	To explore nurse’s experiences of the role of crisis medication in the management of terminal haemorrhage in patients with advanced cancer	Qualitative interviews. Thematic analysis	Terminal haemorrhage is a rapid event and there is often no time for crisis medication to be given or take effect. Determining whether to give crisis medication is challenging and raises anxiety Nurses feel reassured to have medication prescribed even if it may not be used or has time to take effect	M H L – M
Kemp et al.^42^	United Kingdom	Patients registered with 12 GP surgeries in one county	To evaluate the prevalence and impact of anticipatory prescribing on home death/utilisation of healthcare in the last month of life	Retrospective case note review. Statistical analysis	Anticipatory prescribing was in place for 16% of predictable deaths in a 1-year period: levels of usage varied widely between GP surgeries Patients living at home were less likely to have drugs prescribed than those in care homes The use of anticipatory prescribing was associated with an increased chance in home death – however, a causal association was not demonstrated Anticipatory prescribing use was also associated with decreased risk of hospitalisation in last month of life, and increased GP contact in both care home and community residents	M M M – M
Owen et al.^43^	United Kingdom	550 patients who died in 19 nursing homes	Review of care since the GP surgery–based MDT took over medical and pharmacological care of the nursing homes	Retrospective notes review. Statistical analysis	Anticipatory prescribing frequency varied across the nursing homes: 3 nursing homes had it in place for 62% of deaths, and 3 nursing homes had it in place for only 28% of deaths Less than a third of patients who were prescribed drugs had them administered Midazolam and morphine were the most commonly used medications There was a clear correlation (r^2^ = 0.64) between the proportion of patients prescribed anticipatory medications and the proportion of patients dying at the home instead of in hospital There was no correlation between administration of anticipatory medications and place of death	M M M – M
Wilson et al.^20^	United Kingdom	575 nurses working in two regions: 231 nursing home nurses; 151 palliative care nurses; 193 district nurses	To gain insight into the roles and experiences of a wide range of community nurses in end-of-life medication decisions	Staff survey. Descriptive statistics. Thematic analysis of free-text comments	Responses suggest anticipatory prescribing is a widespread practice Where patients’ age categories were reported (n = 412), 63.8% (n = 263) were said to be aged 70 or over A primary cause of death was provided for 434 patient cases and in 79.3% of these, cancer was reported by nurses as the registered cause of death Decision to prescribe often dictated by the nurses rather than the GP Facilitators to prescribing: nurses reported working well with GPs and perceived that they had good access to the medications needed; 79.2% of nurses reported that they ‘infrequently or never’ found doctors reluctant to prescribe anticipatory medication Barriers to prescribing: anticipatory prescriptions being incorrectly written up by doctors; 8.6% of nurses said they ‘always or frequently’ experienced significant difficulties in obtaining the anticipatory drugs Nurses reported that the anticipatory medications successfully controlled those symptoms they were intended to relieve in 89.6% of the patient cases they recalled Midazolam was the drug most commonly reported to have been used in the last month of the patient’s life Nurses felt they were responsible for assessing the patient’s response to drugs	M M M – M
Addicott^44^	United Kingdom	11 healthcare professionals working in two surgeries: 8 GPs; 1 practice nurse; 2 community nurses	To identify challenges and examples of good practice in providing good-quality end-of-life care in general practice	Case study using qualitative interviews. Qualitative analysis	GPs happy to prescribe anticipatory drugs to cover out of hours periods Prescribing considered a significant responsibility as accountable for use/misuse Concerns around large amounts of medication left in home without supervision	L H L – M
Amass and Allen^6^	United Kingdom	23 patients in the community across one region	To evaluate an anticipatory medication pilot	Audit of care. Descriptive statistics	23 anticipatory prescribing kits issued and 16 (70%) were used The intervention was well received by nurses, patients, and carers None of the 16 patients required admission to a hospital or hospice for end-of-life symptom control The net cost of wasted medicines was about £10 per patient	L M M – M
Ashton et al.^33^	United Kingdom	13 care staff working in four care homes and one NHS mental health ward	To assess the effects of the Gold Standards Framework and LPC on the experience of staff	Qualitative focus group. Analysis not stated	Staff acknowledged the difficulties for GPs in anticipatory prescribing, particularly relating to: pain management, the experience of the GP and their understanding of advanced dementia, the reluctance to prescribe diamorphine Staff felt these difficulties would resolve as the GP developed a trusting relationship with them	L H L – M
Ashton et al.^34^	United Kingdom	200 healthcare professionals working in four care homes and one NHS mental health ward	To determine the effects of introducing Gold Standards Framework and LCP from the perspectives of staff involved in the care of older people with dementia	Case study using mixed methods: interviews, focus groups, survey of staff. Analysis not stated	Anticipatory prescribing was viewed as a key element in the management of pain and other distressing symptoms	L H L – M
Bullen et al.^45^	Australia	8 community palliative care nurses. 43 community palliative care services	To conduct a survey of a local service to examine views on medication management before and after the implementation of an anticipatory prescribing kit and to conduct a nationwide prevalence survey examining the use of anticipatory prescribing kits	Quantitative single-arm intervention study with pre- and post-questionnaires in a community specialist palliative care service. Nationwide prevalence survey of the use of anticipatory prescribing kits in Australia	88% of nurses reported the implementation of the anticipatory prescribing kits had improved patient outcomes The administration of anticipatory medications was highly variable and usually occurred when the patient entered a deteriorating or terminal phase of care In the majority of instances where kits were used, the medications were perceived to have met patients’ needs The majority of services surveyed reported they did not use anticipatory prescribing kits Most participants from services who did not utilise anticipatory prescribing kits believed that they could improve patient care Having access to the low-cost kits was perceived to help avoid unnecessary crisis hospital admissions	L M M – M
Harris and Nobel^46^	United Kingdom	152 community, hospice and hospital palliative care teams across the United Kingdom	To explore current practice in the management of terminal haemorrhage by palliative care teams in the United Kingdom	Survey with open and closed questions. Descriptive statistics	Midazolam was the most commonly used crisis medication although there is a large variation in the dose of this and other drugs used Unclear role of crisis medication, as patients often die before they can be given or take effect	M M L – M
Kinley et al.^47^	United Kingdom	319 residents who died in 38 nursing homes taking part in an end-of-life programme	To identify the prescribing practice for symptom control in the last month of life for residents dying in nursing homes	Retrospective notes review. Descriptive statistics	37% of residents had anticipatory prescribing in place at the time of death	M M L – M
Lawton et al.^48^	United Kingdom	58 community nursing teams in one county	To audit staff awareness of an anticipatory prescribing scheme	Audit of practice. Descriptive statistics. Grouping of free-text comments received	The majority of patients issued drugs were diagnosed with a malignancy (n = 43) Difficulty in predicting right time to prescribe anticipatory medications Having prescribed medication available in the home was perceived as reassuring for families Barriers to prescribing: patient not wanting drugs in home; professionals not thinking about anticipatory prescribing; GPs declining to consider anticipatory prescribing The costs of prescriptions were estimated to be to £22.12 per patient A significant amount of medicines went unused, but 77% of boxes issued had at least one drug used	L M M – M
Wowchuk et al.^49^	Canada	457 patients in one region	To evaluate the use of a anticipatory prescribing kit	Service evaluation based on complete data collection forms from accessed anticipatory prescribing kits. Statistical analysis	Pilot project issuing 457 patients with anticipatory prescribing kits over a 5-year period Majority of patients in pilot had cancer (8.5% non-malignant) 293 kits were both placed in patients’ homes and accessed The mean survival from the time the kit was open until the time the patient died was 4.54 days Home death rate much higher in those participating in the pilot medication kit scheme compared to the home death rate for the overall programme 79%–88% home death rate for those who used the kit; 60% home death rate for those who had the kit placed but did not use it; 25%–29% home death rate for those not in the pilot	L M M – M
Dale et al.^13^	United Kingdom	995 surgeries in England and Northern Ireland: those returning baseline and follow up questionnaires	To identify factors associated with the extent of change in processes that occurred in practices in the year following adoption of the Gold Standards Framework	Quantitative uncontrolled observational cohort study with pre-post questionnaire. Statistical analysis	48.9% of surgeries had a procedure for anticipatory prescribing at baseline 82.3% of surgeries had a procedure for anticipatory prescribing a year later	M L L – L
Hardy et al.^50^	Australia	20 patients in one nursing home (as part of a study looking at four hospitals, three hospices and one nursing home)	To evaluate the care of patients who died in institutes in Queensland	Retrospective notes review. Descriptive statistics	Few Patients in the nursing home were prescribed drugs in anticipation of symptoms (no numbers given)	L M L – L
Healy et al.^14^	Australia	76 family carer questionnaires. Focus groups with 26 nurses	To evaluate of the effectiveness of an education package that supports laycarers of home-based palliative patients to manage breakthrough subcutaneous medications used for symptom control	Mixed methods: single-arm intervention study with two post-intervention questionnaires for family carers. Focus groups with nurses	In Australia laycarers, mostly family members may be required to administer subcutaneous medications Family carers found the package was useful and enabled them to deal confidently with symptoms arising in the home-based palliative patient Nurses were uncertain in when to train family carers in the patient’s trajectory Contention between nurses on if it is safe or appropriate for family carers to give drugs	L M L – L
Jamal et al.^51^	United Kingdom	GPs and community nurses (numbers not stated) working in one county	To evaluate the awareness of network guidelines along with the prescribing and usage ratios of anticipatory prescribing kits	Service evaluation. Descriptive statistics	90% of GPs responding indicated that they had prescribed anticipatory prescribing kits 69% of GP’s stated prescribing was influenced by access to anticipatory prescribing information, and 75% stated that levels of confidence impacted on decision-making 55% of GPs respondents indicated that prescribing was influenced by concerns about misuse of drugs 41% of GPs indicated that cost was a factor The recommended network guidelines for 2–3 days’ supply of anticipatory medications costs £30.26 per patient	L M L – L
Lawton et al.^52^	United Kingdom	181 after death reviews with home staff in 56 nursing homes and 25 care homes	To describe factors that promote a ‘good death’ in care homes	Qualitative interviews. Qualitative analysis.	Nursing home staff felt having anticipatory medications in place gave reassurance to residents, staff, and relatives	L M L – L
Lee et al.^53^	United Kingdom	5 informal carers in one county	To audit the feasibility of the policy and practice of informal caregivers administering subcutaneous medication	Audit of care. Reporting on informal carers comments	Informal carers gave injectable anticipatory medications, with nurse support and training All informal carers stated that, if required, they would administer subcutaneous injections again to a family member	L M L – L
Mathews and Finch^54^	United Kingdom	10 patients in one nursing home. Reflective group with nursing staff (number not stated)	To evaluate the impact of implementing the LPC in a nursing home	Audit of patient notes and a reflective group discussion with nurses on implementing the LPC. Analysis methods not stated	GPs prescribe anticipatory medications and nursing home staff judge when to administer drugs Barriers to prescribing: nurses reported GPs reluctant to prescribe diamorphine to opioid naive patients Facilitators to prescribing: GPs familiarity with anticipatory prescribing practice Some nurses worried about administering injectable opioids and felt uneasy when a patient died within hours of an injection	L M L – L
O’Loghlen and Baines^55^	United Kingdom	295 service evaluation forms from 83 GPs surgeries in one county	To evaluate an anticipatory prescribing scheme	Service evaluation. Descriptive statistics	Perception that the scheme offered peace of mind for patients and relatives The information gathered from the completed forms suggested that 121 admissions to hospital or hospice were prevented	M L L – L
Lee and Headland^56^	United Kingdom	2 patients in one county	To report on the feasibility of relatives giving subcutaneous injections	Descriptive case reports from a nurse perspective. Description of care received	Reports on two cases where family carers gave injectable anticipatory medication following training Accounts that the family carers felt this was acceptable and helped with providing effective symptom control at home	L L L – L

Care home: a community residence without trained nurse on site; nursing home: a community residence with trained nurses on site; GP: family doctor; H: high; M: medium; L: low.

Quality of the evidence was assessed using Gough’s Weight of Evidence framework:^30^ (A) coherence and integrity of the evidence in its own terms; (B) appropriateness of the study design in answering the review questions; (C) relevance of the evidence for answering the review questions; and (D) overall assessment of the quality and relevance of the study, derived by combining judgements (A), (B), and (C).

## What is current practice?

Few studies investigated the frequency of anticipatory prescribing in the community: these were primarily limited to the United Kingdom, and patient samples do not accurately represent the general population. ^38,40,42,43,47,50^ Reported figures varied greatly across studies which may relate to differences in study design, context, and denominators used.^38,40,42,43,47,50^ A study of 12 GP practices in one UK county reported that anticipatory prescribing occurred in 16% of all predictable deaths in the community (home or care home).^42^ By contrast, a retrospective case note review of 150 consecutive deaths managed by a specialist palliative care team indicated that 63% of the sample had anticipatory prescribing in place at the time of death.^40^ One Australian nursing home study reported a low rate of anticipatory prescribing but provided no figures.^50^ Three retrospective studies in UK nursing home settings reported anticipatory prescribing rates varying from 37%,^43^ 28%–62%,^47^ to 13–100%.^38^ Although the data are limited by inadequate definitions of anticipatory prescribing,^43,47^ patients at home or in care homes appear less likely to be prescribed drugs than those in nursing homes.^38,42,43,47^ Surveys of community healthcare professionals suggest that anticipatory prescribing is widespread in the United Kingdom.^13,20^

There is wide variation in the timing of anticipatory prescribing prior to death, ranging from a few days^3,49^ to several weeks.^3,5,40^ Difficulties are encountered in predicting when patients are likely to die^3,24^ with GPs and community nurses frequently recalling situations where drugs were not issued in a timely manner.^3,39,48^ Nurses often initiate the process by alerting the GP to a patient’s changing condition and requesting an anticipatory prescription.^5,20,24,37,49^ One study reported nursing home staff would request anticipatory prescriptions weeks ahead of need to mitigate the difficulty of timely GP reviews.^5^

Decisions regarding which anticipatory medications are issued are often shared between GPs and nurses.^24,37^ In most cases, only the GP can issue the prescription: the small number of UK nurse-prescribers still prefer to share decision-making with the GP.^24^ There is considerable variability in the terminal symptoms prescribed for and anticipatory drugs prescribed. There are very limited data about anticipatory drugs prescribed.^20,38,55^ One study,^38^ rated as medium quality, indicates that there was variability in the number and type of terminal symptoms prescribed for across eight nursing homes; 54% of patients had at least one drug prescribed (most commonly for pain and least commonly nausea and vomiting), but only 15% had drugs prescribed for all four recommended indications. A local service evaluation,^55^ rated as low quality, lists the most commonly prescribed drugs but did not provide frequencies.

There is limited literature concerning the relationship of anticipatory prescribing to diagnosis. Cancer was predominant in two studies of anticipatory medication kit implementation^48,49^ (84% and 91.5%), with 79% of community nurses reporting their last experience of anticipatory prescribing was with cancer patients.^20^ Conversely, one retrospective study of 150 consecutive deaths under a community specialist palliative care service found that anticipatory medications were in place at the time of death for 78% of non-cancer deaths (n = 50) but only 55% of cancer deaths (n = 100).^40^ No other data are provided to allow assessment of the comparability of these diagnostic subgroups. Anticipatory medication timing decisions are perceived to be more challenging in the less predictable dying trajectories of non-cancer illnesses.^3^

The literature concerning the use of anticipatory medications after prescription is also limited. Use in nursing homes appears to be less common: one retrospective study reported that ‘less than a third’ of patients required the administration of prescribed medications,^43^ and a qualitative study of nursing home nurses reported that very few dying patients required the administration of prescribed drugs.^5^ Much higher proportions of use have been reported at home, ranging from 70%–77%.^6,48^ The sedative anxiolytic midazolam is identified in three studies as the most frequently administered drug.^20,43,46^ However, two of these studies^20,46^ relied on healthcare professionals recalling the drugs they gave and did not detail actual practice.

The literature suggests that decision-making concerning anticipatory medication administration is often undertaken by nurses without consultation with a doctor.^5,19,20,45,54^ In some situations, a range of doses are prescribed on drug charts, allowing nurses discretion on the dose administered.^19^ In contrast, one Canadian study reported nurses to have a less independent role, needing to gain authorisation from a doctor before administering the drugs.^49^ UK nurses identify four conditions that all need to be met before they administer medication: symptoms are irreversible and due to the dying phase; inability to take oral medication; patient consent where possible; and decisions made independent of influence from family carers.^19^ Nurses often work in pairs when making this assessment or check their decisions with nursing colleagues.^19^ In some areas, family carers have been trained to assess symptoms and give injectable drugs, with or without direct clinical guidance, in Australia^14,22,23^ and United Kingdom.^53,56^

## What are the attitudes of patients?

No studies have investigated patients’ experience of or views towards anticipatory prescribing. One audit,^6^ rated as medium quality, and one service evaluation,^55^ rated as low quality, report anticipatory prescribing to be well received by patients. Both studies were based on practitioner interpretations of patient views rather than patient self-reports.

## What are the attitudes of family carers?

Family carer attitudes have been explored within studies of initiatives to train them to administer anticipatory medications,^14,22,23,56,53^ a context which does not reflect standard practice in most countries. Five UK and Australian studies, of low to medium quality, reported that family carers selected for participation in initiatives found the experience of administering anticipatory medications to be acceptable,^14,22,23,56,53^ although an unreported proportion in one study felt overwhelmed by this expectation.^22^ Family carers reported that anticipatory medications were beneficial to patient comfort^14,22,23^ and enabled patients to remain at home until death.^22,56,53^ One Australian study, of medium quality, reported on family carer administration of anticipatory medications in the context of limited access to trained nurses. Family carers felt they had no option but to administer drugs, were uncertain about the timings of medications, and feared causing an overdose or hastening death.^23^ If symptoms remained uncontrolled post drug administration, family carers felt disempowered and distressed.^23^ All five studies reported only on the attitudes of family carers who were willing to take on the role of administering drugs. No studies have investigated the experience of family caregivers when not involved in administering medications, which is standard practice in most countries.

## What are the attitudes of community healthcare professionals?

The range of views of healthcare professionals towards anticipatory prescribing are reported in 21 studies of community, palliative care, and nursing home nurses, care home staff, pharmacists, GPs, and palliative doctors in limited geographical areas (3 rated as high quality, 13 as medium quality, and 5 as low quality).^3,5,14,19,20,24,33–37,39,41,44–46,48,51,52,54,55^ The majority of the studies focussed on the views and experiences of nurses.^5,19,20,24,35,36,41^ Only two studies explored the views of GPs in detail.^3,37^ The views of emergency ambulance paramedics have not been studied.

These studies suggest that healthcare professionals’ views are largely positive towards anticipatory prescribing. GPs and nurses believe it offers reassurance to patients, family carers, and healthcare professionals; provides timely and effective symptom control; and helps prevent crisis hospital admissions.^5,19,24,34,39,44,48,46,52,55^ The one exception is in terminal haemorrhage when specialist palliative care doctors and nurses believe anticipatory prescribing has limited value, as patients often die before medication can be given or take effect.^35,36,46^

Facilitators of successful anticipatory prescribing are identified in several studies. GPs and nurses generally report working well together;^5,20,24^ partnership is perceived to be vital, with trust between the two parties, mutual respect for each other’s expertise and ease of access to each other essential.^3,24,33,37,39^ GPs who are familiar with end-of-life drugs appear to be more confident about anticipatory prescribing,^24,37,51,54^ finding it easier to prescribe for patients they have known for some time,^3^ and appear to be more likely to prescribe in a timely fashion when receiving regular updates from nurses about a patients’ changing condition.^24^ The development of a rapport with patients and their families is perceived to enable sensitive anticipatory prescribing conversations to take place at an appropriate time.^3,24^

Negative healthcare professional views were also articulated in several studies. GPs are wary about the safety of prescribing strong injectable forms of medications ahead of need, since they are accountable for drug errors or misuse.^3,24,44^ Prescribing decisions were perceived to be harder when the GP does not know the patient’s situation well or there are concerns about possible drug misuse within the home.^3,51^ GPs also express concern about the cost of unused medications.^3,5,51^ Despite these potential barriers, nurses perceive that only a small proportion of GPs are reluctant to prescribe anticipatory medications.^20,24,37,41,48^

The administration of anticipatory medications also raises safety concerns for nurses. They do not want to administer the drugs unless it is clear that the patient is dying, and are conscious of the need to balance the achievement of effective symptom control with the avoidance of over-sedation.^19^ If a patient dies soon after drug administration, particularly of opioids, less experienced nurses worry that the ‘last injection’ may have hastened their death.^5,19,54^ Some nurses think it too burdensome on family carers to train them to administer the injectable drugs.^14^

## What is its impact on patient comfort and symptom control?

Evidence of the impact of anticipatory prescribing on comfort and symptom control is limited to three observational audits and surveys of low to medium quality, none of which used symptom assessment scales. No intervention trial of clinical effectiveness has been conducted to date. One small-scale audit of family carer administration (n = 5) found carers to report that their administration of anticipatory medications had facilitated a peaceful death at home.^53^ One large-scale survey of palliative care, community, and nursing home nurses found 89.6% to report that anticipatory prescribing had helped provide successful symptom relief in the cases they recalled.^20^ Similarly, in a very small pre–post implementation study, 88% (n = 7) of surveyed palliative care nurses reported improved outcomes following the introduction of anticipatory prescribing.^45^

## Is it cost-effective?

The literature to date suggests that anticipatory prescribing is a low-cost intervention when compared to the cost of an inpatient hospital or hospice stay.^51^ The typical cost of supplying 2–3 days’ medication to cover the symptoms of pain, nausea and vomiting, agitation, and breathlessness in the United Kingdom is between £22.12^48^ and £30.26 per patient.^51^ The net cost of unused prescribed medications is estimated to be between £10^6^ and £14.61^48^ per patient. Studies calculating costs derived estimates from incomplete prescribing and administration data,^6,48,51^ limiting the accuracy of findings.

Seven studies of low to medium quality have examined the relationship between anticipatory prescribing and service use. One study of 12 GP practices found anticipatory prescribing to be associated with an increase in GP contacts and a lower risk of hospital admission in the last month of life.^42^ Two small-scale audits^6,53^ and one service evaluation^55^ identified that most patients with an anticipatory medication prescription were not admitted to hospital for symptom control at the end of life. These studies do not report the outcomes for patients not prescribed anticipatory medications. One Canadian service evaluation^49^ and three United Kingdom–based retrospective notes reviews^40,42,43^ identified a positive correlation between anticipatory prescribing and the proportion of patients dying at home. None of these studies accounted for confounding variables, such as the level of support from healthcare services.

## Discussion

### Main findings

This systematic literature review addressed six questions and identified the following findings with regards to anticipatory prescribing in the community:

1. Current practice varies both across countries and within the United Kingdom. There are no reliable data on how often drugs are prescribed or subsequently used in the community. In the United Kingdom, where the majority of data were identified, anticipatory prescribing appears to be widespread. Practice varies in relation to community setting, proximity of prescriptions to death, patient populations, and frequency of administration.
2. No studies have directly investigated the experience or views of patients.
3. Studies of family carers’ attitudes have been limited to evaluations of family carer administration of injectable medications. Although family carers appreciate being able to provide symptom relief, some struggle with the responsibility of assessing patient needs and administering medications. No studies have investigated family carers’ views and experiences of standard UK practice.
4. A large proportion of the published literature focuses on the attitudes and experience of healthcare professionals. GPs and nurses believe that it is reassuring to patients and their family carers, enables better symptom control and helps to prevent crisis hospital admissions. In addition to broadly positive professional experience, GPs and nurses also express safety concerns.
5. Robust evidence of clinical effectiveness is absent, as no intervention trial has been undertaken. Observations from qualitative interviews and retrospective audits suggest it may contribute to symptom relief.
6. Robust evidence of cost-effectiveness is also absent, although it is a low-cost intervention.

In summary, this review demonstrates a paucity of high-quality research concerning anticipatory prescribing. Most studies investigate healthcare professionals’ views or provide limited insights through retrospective case note reviews. No study has prospectively investigated the clinical effectiveness or cost-effectiveness of anticipatory prescribing. Most studies were limited to single sites, evaluated new initiatives or had selected participants, limiting their generalisability.

### What this review adds

This review brings together the diverse literature in regard to anticipatory prescribing, clarifying the current knowledge base and the priority areas for future research.

Current practice is based primarily on GPs’ and nurses’ perceptions and experiences that anticipatory prescribing offers reassurance to patients and family carers and provides effective symptom control in the home setting.^5,19,24,33,34,39,44,48,52,55^ Although the rationale for this practice appears strong intuitively, it is unwise to base end-of-life care practice on healthcare professionals’ views alone. The views of patients and family carers must also be taken into account;^57^ some may view anticipatory prescribing as an unwelcomed indicator of impending death.^24^ Concerns have been raised that prescribing and administration can be paternalistic or service driven rather than tailored to patients’ wishes.^7,58^ Having medication at home places a significant responsibility on family carers to assess symptom control and decide when to request a healthcare professional to administer drugs.^57,59,60^ This responsibility is much greater when family carers are expected to administer injections:^14,22,23,53^ some worry that this might hasten death.^22,23^ Conversely, many family carers value being able to do something to relieve pain and distress.^22,23,61^ Patient and family carer views and experiences of anticipatory prescribing need urgent investigation.

It appears that anticipatory prescribing policies and practice are running ahead of the evidence base. There is a lack of robust evidence for its clinical effectiveness in optimising symptom control and in preventing crisis hospital admissions, alongside the lack of high quality evidence of patient and family carer experience and views. The recent call in UK end-of-life care guidance for a cluster-randomised control trial^7^ may be challenging in countries such as the United Kingdom, where anticipatory prescribing is already a widespread and established practice. However, there is a clear need for well-designed clinical trials investigating the intervention’s impact on patients’ symptom control and crisis hospital admissions.

Patient safety concerns were a recurrent theme in the papers exploring the attitudes of community healthcare professionals. There is a potential for drug errors or misuse^3,24,44^ and recent guidance reiterates the risks of prescribing and administration being standardised rather than individualised to a patient’s needs.^7^ When drugs are prescribed in hospital or hospice prior to discharge, this is not always clearly communicated with community healthcare professionals.^62^ When drugs remain in the home for long periods they may no longer be appropriate. There is also the risk that immediate access to medications reduces out-of-hours doctor visits which may disadvantage patients with potentially reversible problems in need of careful medical assessment.^28^ Research investigating the safety of anticipatory prescribing is urgently needed.

### Limitations and strength of the review

This review sought to systematically identify and synthesise the published evidence. Supported by a professional medical librarian (I.K.), the literature search strategies covered nine pertinent databases using the majority of terms used internationally. Journal hand searches and reference and citation searches identified a further eight papers, four of which were not registered on the electronic databases searched.

The review team included published conference abstracts to ensure comprehensiveness, although all abstracts scored medium or low on Gough’s ‘WoE’ framework due to limited information on their methods.

At times, it proved difficult to separate anticipatory prescribing before symptoms arise from reactive prescribing after symptoms occur in papers describing end-of-life care practice.^3,23,41^ Two reviewers systematically applied the definition of ‘the prescribing of injectable medications ahead of need to control terminal symptoms’^11^ and reached consensus by discussion.

The review findings are limited to the United Kingdom, Australia, and Canada, countries whose similar healthcare systems permit synthesis of data. Although anticipatory prescribing is considered good practice internationally, published empirical research from a number of countries is scant and refers largely to the prescribing of orally and rectally administered medications.^9,15–17,60^

## Conclusion

Anticipatory prescribing is a recommended and widespread practice in many countries, despite an inadequate knowledge base. Policy and practice are running ahead of the evidence, based largely on the belief of healthcare professionals that it reassures patients and their family carers, effectively controls symptoms and prevents crisis hospital admissions. The views and experiences of patients and their family carers have not been adequately investigated; nether has clinical effectiveness, cost-effectiveness, and safety. Our research group is planning a programme of research to help address these knowledge gaps

## Supplemental Material

Supplemental_Document_1_Search_strategy_19.10.18 – Supplemental material for Anticipatory prescribing of injectable medications for adults at the end of life in the community: A systematic literature review and narrative synthesis Click here for additional data file. Supplemental material, Supplemental_Document_1_Search_strategy_19.10.18 for Anticipatory prescribing of injectable medications for adults at the end of life in the community: A systematic literature review and narrative synthesis by Ben Bowers, Richella Ryan, Isla Kuhn and Stephen Barclay in Palliative Medicine

## Supplemental Material

Supplemental_Document_2_Data_Extraction_Tool_19.10.18 – Supplemental material for Anticipatory prescribing of injectable medications for adults at the end of life in the community: A systematic literature review and narrative synthesis Click here for additional data file. Supplemental material, Supplemental_Document_2_Data_Extraction_Tool_19.10.18 for Anticipatory prescribing of injectable medications for adults at the end of life in the community: A systematic literature review and narrative synthesis by Ben Bowers, Richella Ryan, Isla Kuhn and Stephen Barclay in Palliative Medicine
