# Supplementary material for: Anticipatory prescribing of injectable medications for adults at the end of life in the community: A systematic literature review and narrative synthesis
Source: Palliat Med. 2018 Dec 4;33(2):160–77. doi: 10.1177/0269216318815796 (PMC6350182; doi:10.1177/0269216318815796)
Supplement: Supplemental_Document_1_Search_strategy_19.10.18 – Supplemental material for Anticipatory prescribing of injectable medications for adults at the end of life in the community: A systematic literature review and narrative synthesis [file Supplemental_Document_1_Search_strategy_19.10.18.docx]

**Supplemental Document 1: Systematic review search strategy**

All strategies were run on 30^th^ May 2017

## Medline via Ovid

Epub Ahead of Print, In-Process & Other Non-Indexed Citations, Ovid MEDLINE(R) Daily and Ovid MEDLINE(R) 1946 to Present

((palliative adj medicine adj kit*) or (liverpool adj care adj pathway*) or ((end adj2 life) adj2 ((care adj plan*) or (care adj pathway*))) or (gold adj standard* adj framework*) or ((prescrib* or prescription* or medicat* or medicine* or drug* or pharma or pharmaceutical* or packet* or pack* or pak* or box* or kit* or (care adj plan*) or (core adj "4") or (core adj four)) adj3 (crisis* or comfort* or anticipate* or anticipatory or anticipation or preemptive or pre-emptive or (just adj in adj case) or PRN or (pro adj re adj nata) or (as adj required)))).ti,ab. and (exp Terminal Care/ or exp Palliative Care/ or exp "Hospice and Palliative Care Nursing"/ or exp death/ or exp Palliative Medicine/ or exp Terminally Ill/ or ((end adj2 life) or ((final* or last*) adj1 (hour* or day* or minute* or week* or month* or moment*)) or palliat* or terminal* or (end adj stage) or dying or (body adj2 (shutdown or shut* down or deteriorat*)) or deathbed).ti,ab.)

## Embase via Ovid

Embase 1974 to 30^th^ May 2017

((palliative adj medicine adj kit*) or (liverpool adj care adj pathway*) or ((end adj2 life) adj2 ((care adj plan*) or (care adj pathway*))) or (gold adj standard* adj framework*) or ((prescrib* or prescription* or medicat* or medicine* or drug* or pharma or pharmaceutical* or packet* or pack* or pak* or box* or kit* or (care adj plan*) or (core adj "4") or (core adj four)) adj3 (crisis* or comfort* or anticipate* or anticipatory or anticipation or preemptive or pre-emptive or (just adj in adj case) or PRN or (pro adj re adj nata) or (as adj required)))).ti,ab. and (exp *Terminal Care/ or exp *Palliative therapy/ or exp *palliative nursing/ or exp *palliative treatment/ or exp *hospice care/ or exp *hospice/ or exp *Terminally Ill Patient/ or exp *dying/ or ((end adj2 life) or ((final* or last*) adj1 (hour* or day* or minute* or week* or month* or moment*)) or palliat* or terminal* or (end adj stage) or dying or (body adj2 (shutdown or shut* down or deteriorat*)) or deathbed).ti,ab.)

## CINAHL via Ebsco

TI ((“palliative medicine kit*”) or (“liverpool care pathway*”) or ((end N2 life) N2 ((“care plan*”) or (“care pathway*”))) or (“gold standard* framework*”) or ((prescrib* or prescription* or medicat* or medicine* or drug* or pharma or pharmaceutical* or packet* or pack* or pak* or box* or kit* or (“care plan*”) or (“core 4”) or (“core four”)) N3 (crisis* or comfort* or anticipate* or anticipatory or anticipation or preemptive or pre-emptive or (“just in case”) or PRN or (“pro re nata”) or (“as required”)))) or AB ((“palliative medicine kit*”) or (“liverpool care pathway*”) or ((end N2 life) N2 ((“care plan*”) or (“care pathway*”))) or (“gold standard* framework*”) or ((prescrib* or prescription* or medicat* or medicine* or drug* or pharma or pharmaceutical* or packet* or pack* or pak* or box* or kit* or (“care plan*”) or (“core 4”) or (“core four”)) N3 (crisis* or comfort* or anticipate* or anticipatory or anticipation or preemptive or pre-emptive or (“just in case”) or PRN or (“pro re nata”) or (“as required”))))

And

TI ((end N2 life) or ((final* or last*) N1 (hour* or day* or minute* or week* or month* or moment*)) or palliat* or terminal* or (“end stage”) or dying or (body N2 (shutdown or shut* down or deteriorat*)) or deathbed) or AB ((end N2 life) or ((final* or last*) N1 (hour* or day* or minute* or week* or month* or moment*)) or palliat* or terminal* or (“end stage”) or dying or (body N2 (shutdown or shut* down or deteriorat*)) or deathbed) or (MH "Terminal Care+") OR (MH "Palliative Care") OR (MH "Hospice and Palliative Nursing") OR (MH "Hospice Patients") OR (MH "Hospices") OR (MH "Hospice Care") OR (MH "Terminally Ill Patients") OR (MH "Death+")

## PsycINFO via Ebsco

TI ((“palliative medicine kit*”) or (“liverpool care pathway*”) or ((end N2 life) N2 ((“care plan*”) or (“care pathway*”))) or (“gold standard* framework*”) or ((prescrib* or prescription* or medicat* or medicine* or drug* or pharma or pharmaceutical* or packet* or pack* or pak* or box* or kit* or (“care plan*”) or (“core 4”) or (“core four”)) N3 (crisis* or comfort* or anticipate* or anticipatory or anticipation or preemptive or pre-emptive or (“just in case”) or PRN or (“pro re nata”) or (“as required”)))) or AB ((“palliative medicine kit*”) or (“liverpool care pathway*”) or ((end N2 life) N2 ((“care plan*”) or (“care pathway*”))) or (“gold standard* framework*”) or ((prescrib* or prescription* or medicat* or medicine* or drug* or pharma or pharmaceutical* or packet* or pack* or pak* or box* or kit* or (“care plan*”) or (“core 4”) or (“core four”)) N3 (crisis* or comfort* or anticipate* or anticipatory or anticipation or preemptive or pre-emptive or (“just in case”) or PRN or (“pro re nata”) or (“as required”))))

And

TI ((end N2 life) or ((final* or last*) N1 (hour* or day* or minute* or week* or month* or moment*)) or palliat* or terminal* or (“end stage”) or dying or (body N2 (shutdown or shut* down or deteriorat*)) or deathbed) or AB ((end N2 life) or ((final* or last*) N1 (hour* or day* or minute* or week* or month* or moment*)) or palliat* or terminal* or (“end stage”) or dying or (body N2 (shutdown or shut* down or deteriorat*)) or deathbed) or ((DE "Terminally Ill Patients") OR (DE "Palliative Care")) OR (DE "Death and Dying" OR DE "Euthanasia" OR DE "Parental Death") OR (DE "Hospice")

## Web of Science

((“palliative medicine kit*”) or (“liverpool care pathway*”) or ((end near/2 life) near/2 ((“care plan*”) or (“care pathway*”))) or (“gold standard* framework*”) or ((prescrib* or prescription* or medicat* or medicine* or drug* or pharma or pharmaceutical* or packet* or pack* or pak* or box* or kit* or (“care plan*”) or (“core 4") or (“core four”)) near/3 (crisis* or comfort* or anticipate* or anticipatory or anticipation or preemptive or pre-emptive or (“just in case”) or PRN or (“pro re nata”) or (“as required”)))) and ((end near/2 life) or ((final* or last*) near/1 (hour* or day* or minute* or week* or month* or moment*)) or palliat* or terminal* or (“end stage”) or dying or deathbed or (body near/2 deteriorat*) or (body near/2 "shut* down") or (body near/2 shutdown))

## Cochrane Library


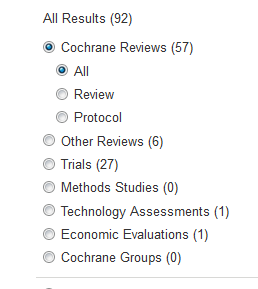


#1 (("palliative medicine kit*") or ("liverpool care pathway*") or ((end near/2 life) near/2 (("care plan*") or ("care pathway*"))) or ("gold standard* framework*") or ((prescrib* or prescription* or medicat* or medicine* or drug* or pharma or pharmaceutical* or packet* or pack* or pak* or box* or kit* or ("care plan*") or ("core 4") or ("core four")) near/3 (crisis* or comfort* or anticipate* or anticipatory or anticipation or preemptive or pre-emptive or ("just in case") or PRN or ("pro re nata") or ("as required")))) 424

#2 ((end near/2 life) or ((final* or last*) near/1 (hour* or day* or minute* or week* or month* or moment*)) or palliat* or terminal* or ("end stage") or dying or deathbed or (body near/2 deteriorat*) or (body near/2 "shut* down") or (body near/2 shutdown)) 19098

#3 MeSH descriptor: [Terminal Care] explode all trees 433

#4 MeSH descriptor: [Palliative Care] explode all trees 1608

#5 MeSH descriptor: [Hospice and Palliative Care Nursing] explode all trees 5

#6 MeSH descriptor: [Palliative Medicine] explode all trees 0

#7 MeSH descriptor: [Death] explode all trees 1869

#8 #2 or #3 or #4 or #5 or #6 or #7 20933

#9 #1 and #8 92

## Social Care Online

"palliative medicine kit" or "liverpool care pathway" or "end of life" and care plan or care pathway or "gold standard framework"

And

"palliative medicine kit" or "liverpool care pathway" or "end of life" and care plan or care pathway or "gold standard framework"

0 hits

"palliative medicine kit" or "liverpool care pathway" or "end of life care plan" or “end of life care pathway” or "gold standard framework"

And

palliative or terminal or death or dying or last or final or "end stage" or “body shutdown” or “body shut down”

Title search: 768 (500 exported

title

medicine or drug or prescription or prescribing or medication or "core 4" or "core four" or packet or pack or box or kit or "care plan"

and

palliative or terminal or death or dying or last or final or "end stage" or body shutdown or body shut down

and

crisis or comfort or comfortable or anticipate or anticipatory or ancticipation or preemptive or pre-emptive or "just in case" or PRN or "pro re nata" or "as required"

All fields 254

## HMIC via Ovid

((palliative adj medicine adj kit*) or (liverpool adj care adj pathway*) or ((end adj2 life) adj2 ((care adj plan*) or (care adj pathway*))) or (gold adj standard* adj framework*) or ((prescrib* or prescription* or medicat* or medicine* or drug* or pharma or pharmaceutical* or packet* or pack* or pak* or box* or kit* or (care adj plan*) or (core adj "4") or (core adj four)) adj3 (crisis* or comfort* or anticipate* or anticipatory or anticipation or preemptive or pre-emptive or (just adj in adj case) or PRN or (pro adj re adj nata) or (as adj required)))).ti,ab. and (exp terminal care/ or exp Terminal nursing/ or exp Terminal illness/ or exp Hospices/ or exp "End of life care"/ or exp Palliative care/ Or exp Death/ or ((end adj2 life) or ((final* or last*) adj1 (hour* or day* or minute* or week* or month* or moment*)) or palliat* or terminal* or (end adj stage) or dying or (body adj2 (shutdown or shut* down or deteriorat*)) or deathbed).ti,ab.)

## King’s Fund

1. Polypharmacy and medicines optimisation

<https://www.kingsfund.org.uk/sites/files/kf/field/field_publication_file/polypharmacy-and-medicines-optimisation-kingsfund-nov13.pdf>

1. seeing the person in the patient

<https://www.kingsfund.org.uk/sites/files/kf/Seeing-the-person-in-the-patient-The-Point-of-Care-review-paper-Goodrich-Cornwell-Kings-Fund-December-2008.pdf>

1. Making our health and care systems fit for an ageing population

<https://www.kingsfund.org.uk/sites/files/kf/field/field_publication_file/making-health-care-systems-fit-ageing-population-oliver-foot-humphries-mar14.pdf>

1. Delivering better care at end of life

<https://www.kingsfund.org.uk/sites/files/kf/Delivering-better-care-end-of-life-Kings-Fund-January-2010-Leeds-Castle-EOLC.pdf>

1. End-of-life care

<https://www.kingsfund.org.uk/sites/files/kf/field/field_document/end-of-life-care-gp-inquiry-research-paper-mar11.pdf>

1. IMPLEMENTING THE END OF LIFE CARE STRATEGY

<https://www.kingsfund.org.uk/sites/files/kf/field/field_publication_file/Implementing-end-of-life-care-Rachael-Addicott-Shilpa-Ross-Kings-Fund-October2010_0.pdf>

1. Improving End of Life Care for Older People

<https://www.kingsfund.org.uk/sites/files/kf/media/Martin-Vernon-Improving-end-of-life-care-for-older-people.pdf>

1. Rapid support close to ‘Care home’ in times of crisis

<https://www.kingsfund.org.uk/sites/files/kf/media/Nicki%20Parry,%20Trinity%20Hospice%20-%20Rapid%20support%20close%20to%20%E2%80%98Care%20home%E2%80%99%20in%20times%20of%20crisis.pdf>

1. Bromley Care Partnership’s Coordination Centre

<https://www.kingsfund.org.uk/sites/files/kf/media/Bromley%20Care%20Partnership%E2%80%99s%20Coordination%20Centre.pdf>

1. Midhurst Macmillan Community Specialist Palliative Care Service

<https://www.kingsfund.org.uk/sites/files/kf/field/field_publication_file/midhurst-macmillan-coordinated-care-case-study-kings-fund-aug13.pdf>

1. NHS England’s National Programme for End of Life Care

<https://www.kingsfund.org.uk/sites/files/kf/media/Jacquie_White.pdf>

1. Making Health and Care services for for an aging population End of Life care

<https://www.kingsfund.org.uk/sites/files/kf/media/Keri-Thomas-Improving-end-of-life-care-for-older-people.pdf>

1. Making end of life care everybody’s business

<https://www.kingsfund.org.uk/sites/files/kf/media/Ilora_Finlay.pdf>

1. reading list - end of life care 2014

<https://www.kingsfund.org.uk/sites/files/kf/field/field_pdf/Library-reading-list-end-of-life-care-Sep2012.pdf>

1. Issues facing commissioners of end-of-life care

<https://www.kingsfund.org.uk/sites/files/kf/issues-facing-commissioners-end-of-life-care-report-september2011.pdf>
