# Supplementary material for: Anticipatory prescribing of injectable medications for adults at the end of life in the community: A systematic literature review and narrative synthesis
Source: Palliat Med. 2018 Dec 4;33(2):160–77. doi: 10.1177/0269216318815796 (PMC6350182; doi:10.1177/0269216318815796)
Supplement: Supplemental_Document_2_Data_Extraction_Tool_19.10.18 – Supplemental material for Anticipatory prescribing of injectable medications for adults at the end of life in the community: A systematic literature review and narrative synthesis [file Supplemental_Document_2_Data_Extraction_Tool_19.10.18.doc]

| **Data Extraction Tool** | |
| --- | --- |
| **Details of publication** | |
| - First author |  |
| - Reference |  |
| **Introduction** | |
| - Aims |  |
| **Study participants** | |
| - Country of study |  |
| - Recruitment |  |
| - Characteristics of participants:   - Number   - Setting (home, hospital etc)   - Age / sex / social class / ethnicity |  |
| **Methods** | |
| - Date of fieldwork |  |
| - Research methods |  |
| - Analysis |  |
| **Key findings relevant to review** | |
| 1) What is **current practice?**  *Who prescribes, for whom, proximity to death?*  *Who administers?*  *Absence of AP?* |  |
| 2) What are the **attitudes of patients** to AP?  *Patients’ acceptance / views* |  |
| 3) What are the **attitudes of family carers** to AP?  *Carers’ acceptance / views* |  |
| 4) What are the **attitudes of community health care professionals** to AP?  *HCPs’ acceptance of AP, concerns, views re use, barriers, facilitators, etc.* |  |
| 5) Evidence for **clinical effectiveness**?  ***Clinical:*** *comfort / symptom control (who reported)* |  |
| 6) Evidence for **cost / cost-effectiveness**?  ***Service use:*** *admission avoidance, place of death, healthcare activity, etc.*  ***Cost****: costs of drugs, admissions, healthcare activity, ect* |  |
| **Author(s) conclusion(s)** |  |
| **Reviewers’ quality assessment of research (Gough WoE)** | |
| **Weight of Evidence A**  Coherence and integrity of the evidence *in its own terms* |  |
| **Weight of Evidence B**  Appropriateness *of form of evidence* for answering review question |  |
| **Weight of Evidence C**  *Relevance of the evidence* for answering review question |  |
| **Weight of Evidence D**  *Overall assessment* of study contribution to answering review question |  |
